# Active sound production of scarab beetle larvae opens up new possibilities for species-specific pest monitoring in soils

**DOI:** 10.1038/s41598-019-46121-y

**Published:** 2019-07-12

**Authors:** Carolyn-Monika Görres, David Chesmore

**Affiliations:** 1 Department of Applied Ecology, Hochschule Geisenheim University, Geisenheim, Germany; 2 Department of Soil Science and Plant Nutrition, Hochschule Geisenheim University, Geisenheim, Germany; 3 Department of Electronic Engineering, The University of York, York, United Kingdom

**Keywords:** Agroecology, Biodiversity, Forest ecology, Data processing

## Abstract

Root-feeding Scarabaeidae larvae can pose a serious threat to agricultural and forest ecosystems, but many details of larval ecology are still unknown. We developed an acoustic data analysis method based on active sound production by larvae (i.e. stridulations) for gaining new insights into larval ecology. In a laboratory study, third instar larvae of the Common Cockchafer (*Melolontha melolontha)* (*n* = 38) and the Forest Cockchafer (*M*. *hippocastani)* (*n* = 15) kept in soil-filled containers were acoustically monitored for 5 min each, resulting in the first known stridulation recordings for each species. Subsequent continuous monitoring of three *M*. *hippocastani* larvae over several hours showed that a single larva could stridulate more than 70 times per hour, and stridulation rates increased drastically with increasing larval abundance. The new fractal dimension-based data analysis method automatically detected audio sections with stridulations and provided a semi-quantitative estimate of stridulation activity. It is the first data analysis method specifically targeting Scarabaeidae larvae stridulations in soils, enabling for the first time non-invasive species-specific pest monitoring.

## Introduction

Melolonthinae, a subfamily of the scarab beetles (Scarabaeidae), comprises of 29 tribes^1^ of which several pose a serious threat to crop and forestry yields all over the world^2–4^. The adult beetles have strong mandibles for mainly eating leaves, however, the most serious damage to trees and agricultural crops is first and foremost caused by the soil-dwelling, root-feeding larvae, also known as white grubs^3^. In Europe, two species of Melolonthinae which are considered as important pest insects are *Melolontha melolontha* (Linnaeus, 1758) (Common Cockchafer) and *M*. *hippocastani* (Fabricius, 1801) (Forest Cockchafer)^2^. These two species are very similar in their biology and are not host plant specific. While *M*. *melolontha* can be found in more open habitats (e.g. pastures, vegetable crops, orchards, vineyards), *M*. *hippocastani* mainly thrives in deciduous forests^5,6^. Currently, these two species occur as pests in Austria, Czech Republic, France, Germany, Italy, Poland and Switzerland, but the available monitoring data is incomplete^2^, and *Melolontha* spp. outbreaks are expected to spread again after a decline in population sizes in the middle of the last century^7^. The reasons behind the recovery of *Melolontha* spp. populations are mainly unknown^7^, but are of serious concern as, for instance, infested forest areas can become more susceptible to droughts and secondary diseases, and forest regeneration can be hindered^5,6,8^.

Strategies to control white grub infestations of *Melolontha* spp. include mechanical (soil-covering nets, ploughing), chemical (pesticides) and biological (*Beauveria* spp., nematodes) measures. These have been applied to various degrees of success during the past century, but until today, there is no generally applicable, environmentally friendly pest control strategy to control white grub infestations at agricultural and forest sites^3,9^. One of the main reasons for this are the difficulties associated with monitoring these soil-dwelling insects^10^. Due to their cryptic lifestyle, white grubs can live for years unnoticed in the soil. The larvae of *M*. *melolontha* and *M*. *hippocastani* live three and four years, respectively, in the soil until they develop into the adult chafer. During this time, the larvae pass three different instars. They only reach pest status if the larval abundance surpasses a certain crop specific threshold^4,8^, for instance, two *M*. *melolontha* larvae m^−2^ are already critical for orchards whereas in meadows *M*. *melolontha* larval abundance of up to 40 larvae m^−2^ can still be tolerable^11^.

The current standard method for monitoring larval abundances and to confirm white grub infestations is to excavate the soil. This type of monitoring is invasive, laborious and time-consuming and cannot be performed at a high temporal frequency^8,10,12^. As a result, *Melolontha* spp. infestations can go unnoticed until severe damage to the vegetation becomes visible, larval abundances can be underestimated, and many details of larval behaviour cannot be satisfactorily explained or predicted yet (e.g. seasonal variations in larval distribution patterns^13^, natural variations in long-term population dynamics^7^, sensory location of preferred host plants^14^, and reactions to different plant substances^6^). Therefore, a stringent way of monitoring is essential for efficient and successful application of already established pest control measures. It can also provide valuable new insights into a target species’ ecology for the development of environmentally friendly pest control measures, such as trap plant selections for integrated plant protection schemes^4,6^.

One way to improve the monitoring of *Melolontha* spp. larvae is the use of acoustics, which has the potential to facilitate non-invasive and real-time continuous soil monitoring^10,15,16^. Soil is a challenging medium for acoustic monitoring due to its heterogeneity. It attenuates sound more strongly than air and plant parts, especially at high frequencies, and sound transmission can vary considerably on a scale of a few centimetres depending on soil properties (e.g. bulk density, organic matter content, soil moisture, root and stone distribution)^15,17,18^. Nevertheless, acoustic sensors inserted into the soil have already been successfully applied for detecting white grub infestations and also for the quantification of larval abundances non-invasively^12,15,17^, but a major challenge is still the correct identification of sounds and the differentiation between pest and nonpest signals^17–20^. The few available acoustic soil studies on Scarabaeidae larvae have only focused on incidental insect sounds (feeding, movement) and species identification still had to be confirmed via soil excavations^15,21^. Several different species belonging to the Melolonthinae can co-occur in soils – not all of them necessarily being regarded as pests^3^ – and even for experts it can be difficult to differentiate the species rapidly in the field when they are still in their larval stage. Especially differentiation of *M*. *melolontha* and *M*. *hippocastani* larvae based only on morphological features alone does not seem possible^22^.

One promising way to overcome this problem is to shift the focus of acoustic monitoring from incidental sounds to stridulations. Stridulations are actively produced sounds for communication created by rubbing together certain body parts^23^. The larval stridulatory organs in Melolonthinae are located maxilla-mandibular and consist of a pars stridens (an area with fine parallel ribs) and a plectrum (sharply confined ridge) which are moving against each other^24,25^. Larvae of many Coleoptera families possess stridulatory organs and morphological descriptions are available for many species^24^, the first description for *M*. *melolontha* larvae stemming already from 1874^25^. In contrast to the vast number of morphological descriptions of stridulatory organs, stridulations themselves have rarely been studied, their ecological meaning is not well understood, and they have never been utilized in soil monitoring programs^19,24,26,27^. However, these sounds seem to have species-specific patterns^26,28^, and thus targeting larval stridulations has the potential to greatly improve pest monitoring by enabling non-invasive, species-specific monitoring with high spatial and temporal coverage. To access this potential for the development of improved soil monitoring tools for *Melolontha* spp. larvae in particular and Scarabaeidae larvae in general, the aim of this study was two-fold: (a) to provide the first description of stridulation patterns of *M*. *melolontha* and *M*. *hippocastani* and to assess their species-specificness, and (b) to develop a data analysis routine for rapid detection and quantitative estimation of scarab beetle larvae stridulations in continuous audio recordings, under the premise of keeping computational costs low to encourage the use of soil acoustics for scarab beetle larvae monitoring.

## Material and Methods

### Cockchafer larvae for acoustic monitoring experiments

Acoustic laboratory measurements were performed with scarab beetle larvae of the species *M*. *hippocastani* and *M*. *melolontha*. These species were chosen due to their important pest status in Europe, but they also serve as model organisms for white grubs in general. Thirty *M*. *hippocastani* larvae (second instar) were excavated in a mixed coniferous forest on sandy soil (Hessisches Ried, Pfungstadt, Germany) in November 2015, and individually kept in small plastic containers (100 ml) with perforated lids in the laboratory in the dark at near constant room temperature (~17 °C). Each container was filled with soil from the excavation site. Approximately every two weeks, carrot slices were added to the containers as food source and the soil sprayed with tap water to keep it from drying out. In the laboratory, larvae shed their exoskeleton once passing from second to third instar in March 2016. In June 2016, 15 larvae were randomly selected for acoustic monitoring in the laboratory. Seventy-five third instar larvae of *M*. *melolontha* were excavated in a meadow on sandy soil (Blaubeuren-Weiler, Germany) in May 2017, and transferred to the laboratory being kept in the same way as the *M*. *hippocastani* larvae. One week after excavation, 38 of these *M*. *melolontha* larvae were randomly selected for acoustic monitoring.

### Acoustic monitoring experiment I

All acoustic measurements were conducted at room temperature (~20 °C) in a plastic box (60 × 40 × 33 cm) insulated with acoustic foam to reduce background noises, a so-called silent box. Detailed instructions for replicating the acoustic sensors described in the following paragraphs can be obtained from David Chesmore. Each larva was acoustically monitored for 5 min by placing one plastic container at a time into the silent box with a low-cost sensor attached to the outside wall of the container. The sensor was self-made based on a piezoelectric transducer (amplified, gain 20). It was connected to an external battery box which, in turn, was plugged into the microphone input of a commercially available audio recorder (TASCAM Linear PCM Recorder DR-05 Version 2, TEAC Europe GmbH, Wiesbaden, Germany). Sounds were recorded in. wav format with an audio sampling rate of 44.1 kHz. Audio recordings of individual *M*. *hippocastani* and *M*. *melolontha* larvae were conducted in June 2016 and June 2017, respectively.

### Acoustic monitoring experiment II

The three most sound-producing *M*. *hippocastani* larvae were selected for a second experiment. An acoustic sensor consisting of a piezoelectric transducer encased in a water-proof, silicone sealed plastic case (length: 21 cm, width: 3 cm, thickness: 0.5 cm) was positioned upright in a glass jar (volume: 2.7 l, height: 24 cm, diameter: 10 cm). Subsequently, the glass jar was completely filled with sandy soil from the original excavation site of the larvae. In the first step of the experiment, one of the three selected *M*. *hippocastani* larvae was placed on top of the soil together with fresh carrot slices as food source. The larva had to burrow itself into the soil. Upon placing the larva in the glass jar, sounds were recorded continuously with the buried sensor for 12 hours. A new audio file was created every 50 min. Two weeks later, the remaining two selected *M*. *hippocastani* larvae were also added to the jar together with fresh carrot slices. A new continuous audio recording was started, this time lasting for 18 hours. Apart from the sensor, the audio recording equipment and the audio sampling rate were the same as in the first experiment. This experiment was performed at the same temperature and with the same silent box used in the first experiment. During the recordings, the silent box was stored in a room with little background noise.

### Manual acoustic data analysis

All raw audio files were bandpass filtered to retain only audio signals between 200 and 5000 Hz, their audio waveforms normalized to a maximum amplitude of −1.0 dB, and any hardware-introduced DC offset removed to centre all audio waveforms on the 0.0 horizontal line^29^. Based on literature and previous experiments, the frequencies filtered out were considered to contain mainly background noise, but not scarab beetle larvae stridulations. Removal of background noise is a standard procedure to improve the performance of subsequently applied data analysis methods^15^.

After pre-processing, each audio file was listened to in real time and visually inspected to manually detect and count all stridulation events. Stridulations with the highest recording quality were selected for detailed inspection of the audio signals’ waveforms and spectrograms for the description of *Melolontha* spp. stridulation patterns. The entire manual acoustic data analysis was performed with the software Audacity 2.1.3^29^.

### Automated acoustic data analysis

**Figure 1:**
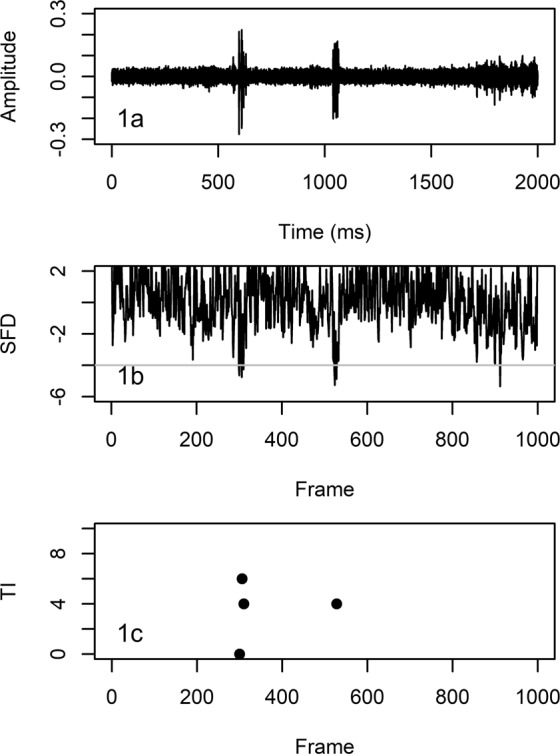
Detection of cockchafer larvae stridulations using fractal dimension analysis (see text for details). (**a**) Audio recording with two stridulations (at ~650 ms and ~1100 ms) and larval moving sounds (from ~1700 ms onwards). (**b**) Summed fractal distance (*SFD*) for every 2 ms (=frame) of the audio recording. Peaks crossing a threshold of −4.0 are first indicators of stridulation events. (**c**) Number of frames between adjacent peaks (*TI*) crossing the threshold in (**b**). A distance of less than 10 frames is indicative of a peak of clusters crossing the threshold in (**b**), and thus a stridulation event.

A data analysis routine for rapid automated detection and quantitative estimation of stridulation events in continuous audio recordings was developed based on the work of Schofield^30^, who was the first to use fractal dimension analysis for the detection of larval activity sounds, specifically larval feeding bites. Fractal dimension analysis focuses on the time domain (i.e. the waveform) of an audio recording, which keeps computational costs low in comparison to other acoustic data analysis methods, and it is amplitude independent, which makes it suitable for environments with low signal-to-noise ratios like soils^30,31^. Fractals can be defined as irregular geometric objects and the aim of fractal dimension analysis is to approximate their shapes, referred to as geometric complexity, through the calculation of scalar values. These scalar values are referred to as fractal dimensions^31^. The waveform of an audio recording is considered as a geometric shape^31^ and the geometric complexity of stridulation events differs from incidental larval sounds (movement, feeding sounds) or background noise (Fig. 1a). The data analysis routine was written with the software R 3.4.3^32^ utilizing the R packages “fractaldim_0.8–4”^33^ and “tuneR_1.3.2”^34^ (see Supplementary Information for a detailed description of the data analysis routine). It was applied to all audio files generated in the second experiment after these files were pre-processed in Audacity as described.

First, the pre-processed audio files were imported into R and sliced into 2 s long sections (*sc*), of which only the amplitude values of the waveforms were extracted for further analysis (Fig. 1a). The next steps were to divide each *sc* into subsections (=frames) with a fixed length (=frame size), and to calculate a fractal dimension (*D*) for each frame (*f*) for converting the geometric complexity of the waveform into a *D* timeseries. Section length and frame size depend on the length of the targeted sounds. The targeted sounds should only take up a small *sc* fraction, thus they can be identified with an outlier detection approach (see following paragraph). Frame size should be about the same size as the targeted sounds or smaller. Smaller frame sizes can increase sound detectability, but processing time per *sc* increases as well. For each *sc*, *D* for the waveform were calculated twice with the madogram estimator^35^ and non-overlapping *f* using a frame size of 88.2 samples (=2 ms) and 176.4 samples (=4 ms), respectively. These frame sizes turned out to be most suitable for appropriately capturing *Melolontha* spp. stridulations by testing different frame sizes on a subset of audio recordings from acoustic monitoring experiment I. Non-overlapping frames were chosen to keep processing time per *sc* as low as possible.

Subsequently for each *f* in *sc*, *D* was converted into a fractal distance (*FD*) by calculating its median deviation from the median (md)^36^:

$$F{D}_{f}=({D}_{f}\mbox{--}{\rm{median}}\,({D}_{sc}))/\mathrm{md}({D}_{sc})$$

The *FD* timeseries with a frame size of 88.2 samples consisted of 1000 samples for each *sc*. The *FD* timeseries with a frame size of 176.4 samples was linearly interpolated to comprise the same amount of samples, and then both timeseries were summed up (*SFD*) (Fig. 1b). The *SFD* timeseries was used for the detection and quantitative estimation of stridulations by applying several vertical and horizontal thresholds. The following thresholds were derived in the same manner as the frame sizes. First, the R function ‘rle’ (=run length encoding) was used to filter out all *f* with *SFD* > −4.0. Second, all *f* were filtered out where *SFD* < −4.0 for more than 2 adjacent *f*. Third, the time interval (in *f*) between the remaining *f* was calculated (*TI*). Clusters of single *f* with *SFD* < −4.0 being spaced apart less than 10 *TI* within the clusters were indicative of stridulation events (Fig. 1c). For each 50 min audio file, stridulation activity (*STRAC*) was automatically estimated by multiplying *TI* 1 to 10 with their respective frequencies and summing up the products.

### Ethical approval

All applicable international, national, and/or institutional guidelines for the care and use of animals were followed.

## Results

### General stridulation patterns in acoustic monitoring experiments I and II

**Figure 2:**
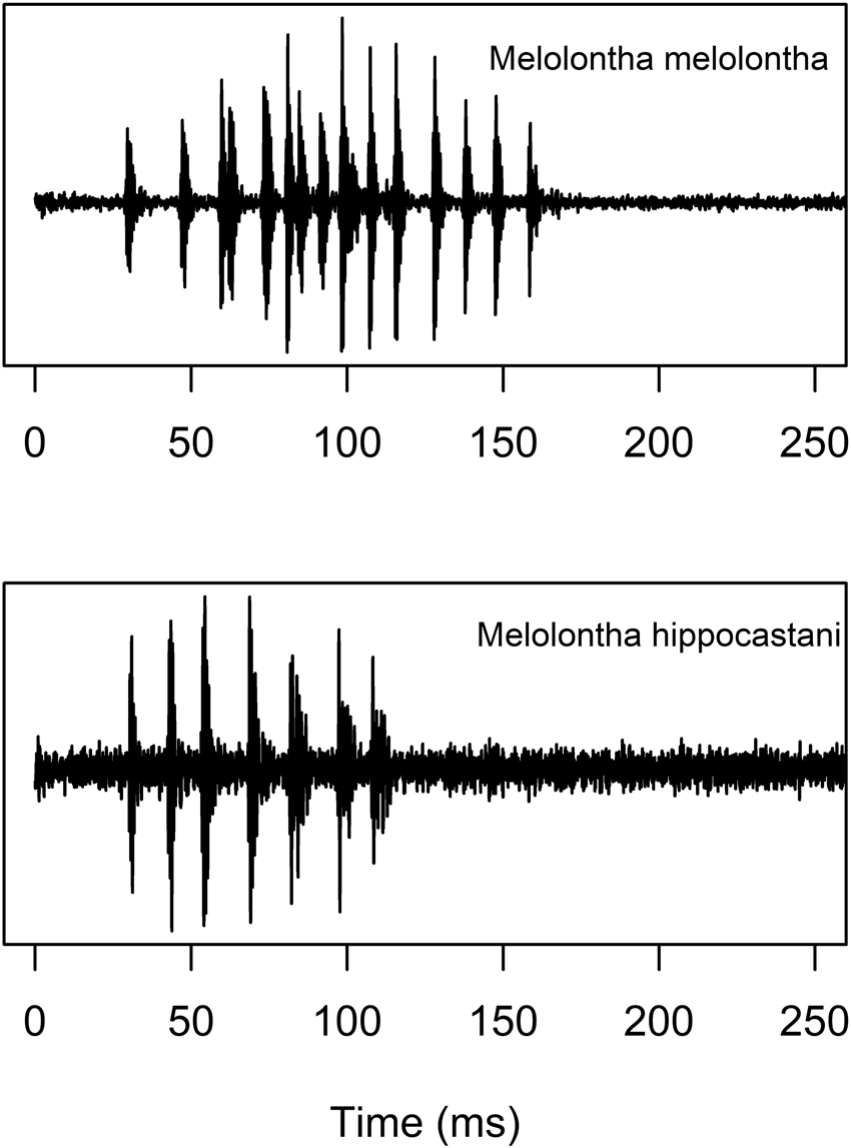
Comparison of acoustic patterns produced by stridulation of larvae (third instar) of *Melolontha melolontha* and *M*. *hippocastani*.

**Figure 3:**
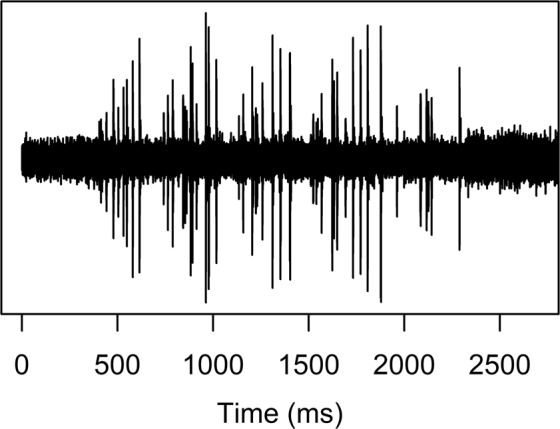
Acoustic pattern produced by stridulation of a third instar *Melolontha hippocastani*.

Stridulations of the two species were easily recognizable and distinguishable by listening to the audio recordings. The common stridulations of *M*. *melolontha* and *M*. *hippocastani* consisted of short bursts of sound (Fig. 2). They were very similar and both peaked at a frequency of ~1700 Hz, however, *M*. *melolontha* stridulations lasted longer than those of *M*. *hippocastani*. Stridulations often occurred in pairs, but repetitions up to 4 times were also recorded. A second type of stridulation which was recorded less frequently from both species consisted usually of 4 (seldom only 2 or 3) repeated patterns with a duration of ~250 ms each and a frequency peak at 3000 Hz (Fig. 3).

### Distribution of stridulation events in acoustic monitoring experiments I and II

During the 5 min manual acoustic screening of individual larvae, only few stridulations were detected. In total, only 5 stridulations from 3 different individuals were recorded from the set of 15 *M*. *hippocastani* larvae. Of the 38 *M*. *melolontha* larvae, 5 individuals were caught stridulating, producing 16 stridulations altogether.

**Figure 4:**
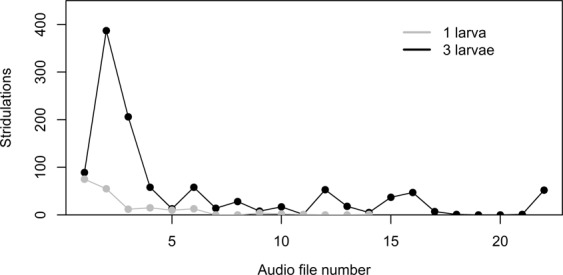
Stridulations manually counted in continuous audio recordings of third instar *Melolontha hippocastani* activities in laboratory soil incubations with 1 and 3 larvae, respectively. Each audio file was 50 min long. For the incubation with 1 larva, only 14 audio files were sequentially recorded.

In contrast to the first experiment, numerous stridulations were observed while manually screening the continuous acoustic monitoring of *M*. *hippocastani* larvae in the second experiment (Fig. 4). The first larva which was placed in the soil-filled glass jar stridulated 75 times in the first 50 min after placement. The stridulation rate dropped to 15 stridulations h^−1^ over the next 4 h and subsequently, the larva almost completely stopped stridulating except for a few single stridulation events. In total, the larva produced 188 stridulations during 12 h of continuous recording. Stridulation activity drastically increased in the soil-filled glass jar after adding 2 more larvae. In the first 2.5 h alone, the 3 *M*. *hippocastani* larvae stridulated 682 times. Afterwards, the stridulation rate levelled below 70 stridulations h^−1^ with periods of high activity alternating with periods of very low activity. Over the course of 18 h of continuous recording, the 3 larvae produced 1100 stridulations.

### Automated acoustic data analysis

For the fractal dimension analysis, the chosen *f* sizes of 88.2 and 176.4 samples were best suited for describing the geometric complexity of stridulation events in the time domain, and thus for detecting them in the amplitude timeseries. For stridulation events, *FD* became more negative in comparison to incidental sounds (movement and feeding sounds, background noise, and interferences). Summing up the two *FD* time series for each analysed *sc* enhanced that effect, separating stridulations from incidental sounds even further along the y-axis. A threshold value of −4.0 was determined to be most suitable for separating stridulations from incidental sounds. Positive S*FD* values were associated with background noise and could be completely disregarded in any further analysis (Fig. 1b).

The S*FD* threshold value of −4.0 filtered out most of the incidental sounds, but for some of them S*FD* also fell below −4.0. However, the short bursts of sounds which made up one stridulation event translated into a S*FD* timeseries in which a cluster of peaks with individual peak widths of 1 or 2*f* and spacing between peaks ranging from 2 to 10*f* passed the threshold (Fig. 1b, c). For larval movement sounds and interferences, S*FD* peaks passing the threshold were usually wider than 2*f*, and not clustered. Thus, including the second and third filter step in the data analysis routine significantly improved its capability for detecting stridulations while ignoring the majority of other sounds regardless of their origin.

**Figure 5:**
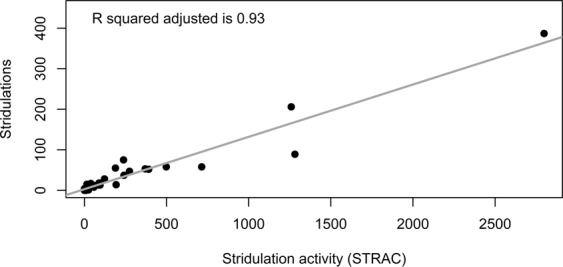
Linear regression of manually counted stridulations on automatically calculated stridulation activity (*STRAC*). Each data point came from a 50 min audio recording. The stridulation data were the same as in Fig. 4, but without differentiation between the numbers of larvae in the soil. Stridulation activity was calculated by multiplying *TI* 1 to 10 (see Fig. 1) with their respective frequencies in each 50 min audio recording and summing up the resulting products.

The data analysis routine based on fractal dimension did not provide a direct count of stridulation events or of sound bursts within single stridulations in an audio recording. The combined 1288 stridulations in the second experiment were spread only over 893 *sc* (=30 min) of the entire audio recordings. Stridulations were directly identified by the data analysis routine in 379 *sc* (42%). 274 *sc* (31%) with stridulations contained only one *SFD* peak passing the −4.0 threshold, no *SFD* peak clusters, and 240 *sc* (27%) with stridulations were not detected. However, the automatically calculated *STRAC* could be used as an estimator for the manually determined stridulation activity (Fig. 5). When excluding the three highest values shown in Fig. 5, the R^2^_adj._ of the linear regression was still 0.72.

## Discussion

In this study, we present, to our knowledge, the first verified audio recordings of larval stridulations from *M*. *melolontha* and *M*. *hippocastani*. The stridulations of the two species sounded similar, but were still distinguishable for a trained listener, with the main difference laying in the overall duration of a single stridulation. Since third instar larvae *M*. *melolontha* are significantly larger than that of *M*. *hippocastani*, and larger stridulatory organs possibly allow longer scraping times, this length difference might have simply been the result of size differences between the larvae of the two studied species. If, indeed, this was the case, a stridulating second instar *M*. *melolontha* might not be distinguishable from a third instar *M*. *hippocastani* in areas where both species co-occur. However, for pest control purposes it is already of great value to have an overview of the distribution of the genus *Melolontha* in the soil. Although we have no stridulation recordings of soil-dwelling Scarabaeidae larvae co-occurring with *Melolontha* spp. yet, it has already been shown for saproxylic Scarabaeidae larvae that stridulations can be used for non-invasive species-specific monitoring^26^.

The ecological meaning of Scarabaeidae larval stridulations is not well understood^24^ although they are mostly interpreted as a territorial defence technique, i.e. stridulating larvae signal their presence to other larvae to avoid competition for resources and to forgo cannibalism^28^. Cannibalism is not uncommon^28,37^ and is also known for *Melolontha* spp larvae. In a laboratory experiment it was observed that *Lucanus cervus* (LINNAEUS, 1758) larvae stridulated much more frequently directly after they were placed in a terrarium than later on^27^. We observed the same stridulation behaviour with *M*. *hippocastani* in our second acoustic monitoring experiment. A likely explanation for this behaviour is that larvae use stridulations to orient themselves in a new environment, but stridulations diminish once the larvae have settled in their new position^27^. Apart from this, it is not known if there are specific times in a Scarabaeidae larval life cycle during which stridulations occur more frequently than in others, if the larvae have a diurnal or seasonal rhythm, or what their complete repertoire of sounds is. In our fast screening of larvae, we only detected very few stridulations, and thus far, only one stridulation of a *M*. *hippocastani* larva has been recorded in the field in undisturbed soil during a survey measurement (data not shown).

To gain a better understanding of Scarabaeidae larval stridulations and to utilise them for species-specific pest monitoring in soils, further laboratory and especially field studies are necessary. One focus of these studies should be the further development of automated sound detection and classification tools. Acoustic monitoring can easily produce large data volumes, whose analysis is very time-consuming if performed manually, and thus presents a bottleneck for widespread application of this monitoring method^38^. We developed the first semi-automated data analysis routine specifically targeting Melolonthinae larval stridulations in soil. The principle idea of this data analysis routine is to consider target sounds in an audio recording as outliers which can be separated from the background noise by simple outlier detection approaches^30,36^. Schofield measured fractal dimensions within a recording as distances from the mean value in multiples of the standard deviation and used a vertical threshold level of 3 to detect larval feeding bites^30^. For larval feeding bites, the *f* size for *FD* calculation should ideally be equal to the length of the targeted event^31^. This is not feasible for *Melolontha* spp. stridulation events which are significantly longer than feeding bites and the likelihood to falsely detect background noise increases with increasing *f* size. Instead, *f* sizes were kept small to target the single pulses within stridulations. That stridulations take up a much larger proportion in a recording than feeding bites also significantly affects the standard deviation around the mean. For the implementation of a vertical stridulation detection threshold, it proved more successful (a) to use the median deviation around the median as a more robust measure of dispersion^36^, and (b) to separate the *FD* of stridulations and background noise further along the y-axis by combining the results of two frame sizes similarly able to capture stridulation pulses. The vertical S*FD* detection threshold separates stridulations pulses and incidental sounds with similar geometrical complexity from any other sounds. Stridulations within this subset can then be targeted by the newly developed horizontal filter, which takes into account the distinct temporal pattern of *Melolontha* spp. stridulations. It is unknown how stable the observed temporal stridulation pattern is, but this uncertainty is accounted for in the data analysis routine by targeting single stridulation pulses instead of entire stridulations.

The fractal dimension-based method is a fast and non-compute-intensive method for pinpointing target sounds in continuous audio recordings in comparison to the spectral profile analysis generally used in acoustic soil studies on Scarabaeidae larvae^21,30^. It can be easily adjusted for detecting different sound types if needed by adjusting *f* sizes and the thresholds for the vertical and horizontal *SFD* filter. For the laboratory experiment, the result of the horizontal filter could be used to calculate a stridulation estimator (*STRAC*), which correlated well with the manually counted stridulations. Furthermore, the number of stridulations clearly increased with increasing larval abundance. If such a relationship between the newly developed stridulation estimator and larval abundances can be verified in the field, it would allow non-invasive species-specific larval abundance measurements with a single acoustic sensor per monitoring plot for the first time. Previous studies using a single acoustic sensor to monitor incidental larval sounds were able to determine with high accuracy the presence or absence of Scarabaeidae infestations, but found only a weak correlation between sound rate and larval abundance^21^. One study managed to predict larval abundances based on incidental sounds by using four sensors at a recording point, but such set-ups are more time-consuming to operate than a single sensor system^17,39^.

The newly developed data analysis routine is simple to use even for non-statistically trained ecologists and pest monitoring practitioners, but it comes with two limitations. It cannot provide an absolute stridulation count, and the detected sounds have to be verified manually by an experienced user. The performance of the fractal dimension analysis ultimately depends on what other sounds are present in the targeted audio recording even when applying a robust measure of dispersion as filter. The geometrical complexity of incidental sounds can vary widely and overlap with the geometrical complexity of stridulations leading to false positives, whereas stridulations can be distorted during transmission through soil in a way that they are not detectable anymore with the chosen filters. These limitations could be overcome by utilising artificial intelligence-based technologies or machine learning algorithms for automated stridulation pattern detection. Such tools have proven very effective and efficient in sound recognition and categorization^38,40^, for instance for acoustic cicada and bird detections^41,42^. However, they come with their own caveats which can discourage pest monitoring practitioners from using them^38^. In addition to higher computational costs, advanced programming or math skills are necessary to further develop and customize the used algorithms^30,38,40^. Furthermore, advanced classification algorithms often need large training datasets which are not available yet for soil-dwelling Scarabaeidae larvae^42,43^. To accommodate the needs of different users, a combination of simple and advanced (semi-)automated sound recognition and classification tools could be a promising way forward in acoustic soil pest monitoring.

In conclusion, this study presents the first stridulation audio recordings of *M*. *melolontha* and *M*. *hippocastani* larvae and demonstrates their applicability for easy identification of these two species. We also designed a new data analysis routine for rapid detection and quantitative estimation of *Melolontha* spp. stridulation events in soil audio recordings based on fractal dimension. *Melolontha* spp. were chosen due to their pest status in Europe, but they also serve as model organisms for white grubs in general. The acoustic data analysis method presented here should be easily transferable to other soil-dwelling Melolonthinae and Scarabaeidae species, providing for the first time the basis for the development of tools for non-invasive, species-specific, and rapid pest monitoring in soils. Acoustic monitoring should not be restricted to incidental sounds, but also include stridulations to make use of its full potential for gaining significant new insights into insect ecology and biodiversity in general, and pest monitoring in particular.

## Supplementary information

Appendix S1 – R code for Melolontha spp. stridulation detection using fractal dimension analysis

## Data Availability

The datasets generated and analysed during the current study are available in the Dryad Digital Repository (10.5061/dryad.2j87692).
